# Periopathogenic bacteria in dental plaque of Congolese patients with periodontitis: A pilot study

**DOI:** 10.4317/jced.54613

**Published:** 2018-03-01

**Authors:** Em Kalala-Kazadi, Jean-Paul Sekele-Issouradi, Jaques Bolenge-Ileboso, Jérôme F. Lasserre, Augustin Mantshumba-Milolo, Hubert Ntumba-Mulumba, Michel C. Brecx

**Affiliations:** 1 PhD student, Unit of Periodontology, Department of Dental Medicine, Faculty of Medicine, University of Kinshasa, Democratic Republic of Congo; 2 Professor, Service of Prosthodontics and orthodontics, Department of Dental Medicine, University of Kinshasa; 3 Associate Professor, Chairman, Unit of Periodontology, Department of Dental Medicine, Faculty of Medicine, University of Kinshasa, Democratic Republic of Congo; 4 Assistant Professor, Department of Periodontology, Université catholique de Louvain, Brussels, Belgium; 5 Associate Professor, Service of Prosthodontics and orthodontics, Department of Dental Medicine, University of Kinshasa; 6 Professor and Chairman of Dental Medicine Department, Service of Prosthodontics and orthodontics, Department of Dental Medicine, University of Kinshasa; 7 Professor, Department of Periodontology, Université catholique de Louvain, Brussels, Belgium

## Abstract

**Background:**

Periopathogenic bacteria play an important role in the etiology of periodontal disease. At present, no study screening for periopathogens in the DR Congo was carried out. The aim of this pilot study was to investigate the prevalence of five periopathogens in Congolese patients with periodontitis and to determine the association between these bacteria.

**Material and Methods:**

Twelve patients (eight women and four men) with a mean age of 45 ± 19 years from those consulted in dental services of two medical centers of Kinshasa from April 2017 to October 2017 were included. Full mouth examination was registered, the probing pocket depth and clinical attachment level were assessed at six sites per tooth. Dental subgingival plaque samples were taken in the deepest pocket per arch in the maxilla and mandible. DNA analysis was performed using DNA-strip technology. The Fisher Exact test and Pearson correlation were used for statistical analysis.

**Results:**

*Porphyromonas gingivalis* and *Tannerella forsythia* were detected at high level of 92%, *Prevotella intermedia* at a rate of 75% whereas *Treponema denticola* was detected in all patients. *Aggregatibacter actinomycetemcomitans* was not detected. Strong associations were found between three bacteria of the red complex and between *T. denticola* and *P. intermedia* (r=1).

**Conclusions:**

This first study investigating periopathogens in subgingival plaque of Congolese with periodontitis demonstrated a high prevalence of the red complex (*P. gingivalis*, *T. forsythia *and *T. denticola*). Associations between different bacteria of this complex were strong.

** Key words:**Association, bacteria, periopathogen, periodontitis, prevalence.

## Introduction

Periodontitis is an infectious and multifactorial disease characterized by loss of the tissues supporting the teeth. Microbes within the dental biofilm initiate the inflammation that can lead to tissues breakdown in the susceptible host (1). The microbial etiology of periodontal disease has been investigated for many years and the role of bacteria species has been extensively documented during the last decades (2-4). The arrival of molecular techniques has led insights in this area and periodontal microbiology has been an area of intense research for decades (5).

From the study by Socransky *et al.* 1998 (3), *P. gingivalis*, *T. forsythia* and *T. denticola*, the so called “red complex”, has been reported to be strongly associated with clinical parameters of chronic periodontitis (6-8). Also *A. actinomycetemcomitans* has been reported as a key pathogen in aggressive periodontitis and especially in localized aggressive periodontitis (9-11). From the non-specific plaque hypothesis to the specific one and more recently the ecological hypothesis, the role of microorganisms in the etiology of periodontal disease remains of great interest.

The majority of these studies were carried out in Europe and in the United States of America. Only scarce studies were carried out in Africa (11-13). Indeed, the microbiota associated with periodontitis shows important geographical particularity (14) as well as differences among different ethnic or racial groups (15,16).

The objective of this pilot study was to assess, using molecular technique, the prevalence of three target members of the red complex (*P. gingivalis*, *T. forsythia* and *T. denticola*), *Aggregatibacter actinomycetemcomitans* and of one member of the orange complex, *Prevotella intermedia* in subgingival plaque of Congolese patients with periodontitis and to determine the association between these different bacteria.

## Material and Methods

-Selection of the study group and clinical examination

This pilot study included twelve patients (eight women and four men). Their age varied between 14 to 72 years old. The mean age was 45 ± 19 years. They were selected among the patients consulted in the dental services of two medical centers, the Ngaliema and Boyambi clinics between April 2017 and October 2017 in Kinshasa. This study was approved by the ethics committee, school of public health of the University of Kinshasa, approval number ESP/015/2017. An informed consent was obtained from each patient and pertinent information concerning the study was explained to all of them.

To be part of this study, these patients should have at least 20 teeth and aged twelve years and older, and if they had at least a probing pocket depth (PPD) of 5mm and a clinical attachment level (CAL) of 4mm on at least two teeth, one in the maxilla and another in the mandible. Patients who had taken antibiotics for the last six months and those under periodontal treatment or who had underwent periodontal treatment for the last six months were excluded.

Full mouth examination was made using a periodontal probe (Hu-Friedy, PCP 10, Chicago, Illinois, USA), all teeth were examined excluding the third molars. Also excluded were erupting teeth, supernumerary and partially impacted teeth. The PPD and CAL were assessed at six sites per tooth. The plaque index (PlI) and bleeding on probing (BoP) were also measured. All measurements were performed by a single trained and calibrated examiner (E.K.K).

-Microbial sampling

The subgingival biofilm samples were obtained from the two deepest pockets per patient, one in the upper arch and another in the lower arch previously chosen during the clinical examination.

The sampled site was isolated using sterile cotton rolls. The supragingival plaque was first removed with a sterile gauze and then a sterile Gracey curette. A sterile paper point was introduced into the pocket and left in place for ten seconds. This paper point was then removed and placed immediately in the Eppendorf plastic tube. The Eppendorf tubes closed were placed in the micro-IDentᴿ kit provided by Biocentric Laboratory (Hain Lifescience, Nehren, Germany).

-DNA analysis 

The plaque samples were sent in France where the PCR analysis was performed by the Biocentric laboratory (Bandol, France). The technique used in this laboratory is based on semi-quantitative PCR using DNA-strip technology (Hain Lifescience, Nehren, Germany). The threshold for bacterium detection is 10³ for *A. actinomycetemcomitans* and 104 for *P. gingivalis*, *T. forsythia*, *T. denticola* and *P. intermedia*. In summary, DNA was extracted from the specimen by thermolysis at 95◦C. Then, DNA was selectively amplified in a subsequent PCR reaction. After that, the amplified DNA was denaturized followed by hybridization on strip with specific probes.

-Statistical analysis 

The statistical analysis was made using the Statistical Package for Social Sciences software (SPSS 20.0., Chicago, IL, USA). Associations between periopathogenic bacteria were assessed by the Fisher Exact test and the Pearson correlation.

## Results

The majority of participants (58%) had never been treated previously by a dentist and 50% had a high school level whereas the remaining 50% had middle to low school levels. Only 8% of them were current smokers ( Table 1).

**Table 1:**
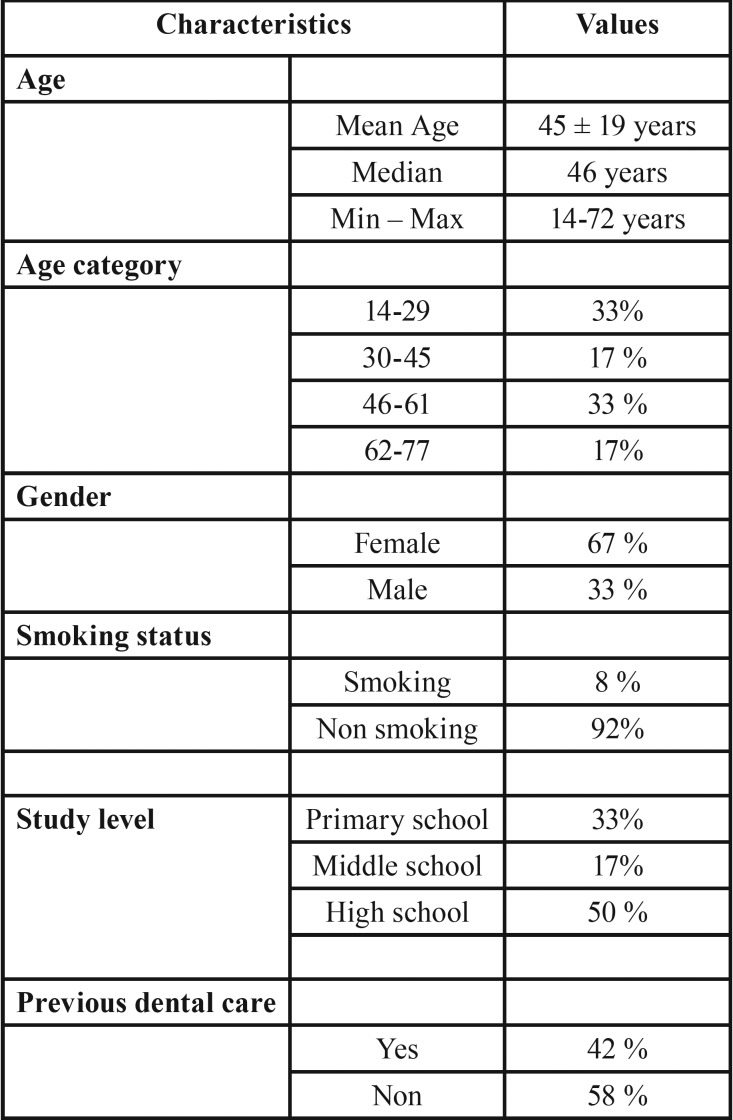
Characteristics of the study population.

 The mean BoP for participants was 42% ± 25 and the mean PlI was 1.0 ± 0.5. The mean number of teeth per patient with a PPD of 6mm and over was 1.5 ± 2.1; that with a PPD of 4 to 5 mm was 9.8 ± 4.1 ( Table 2).

**Table 2:**
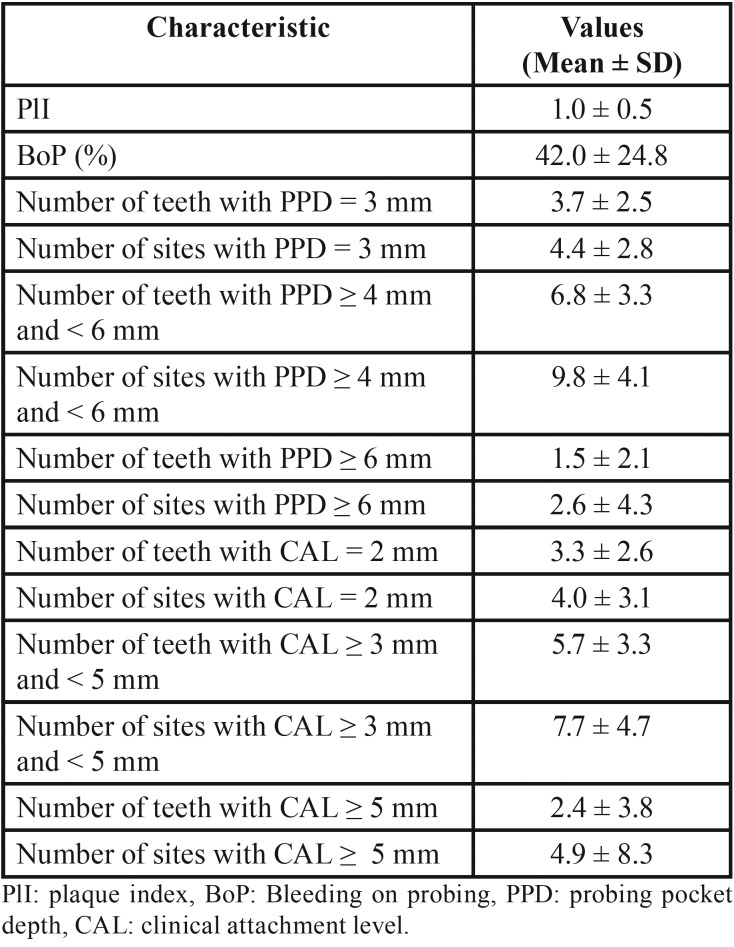
Extent and severity of periodontitis in the study population.

**Figure 1:**
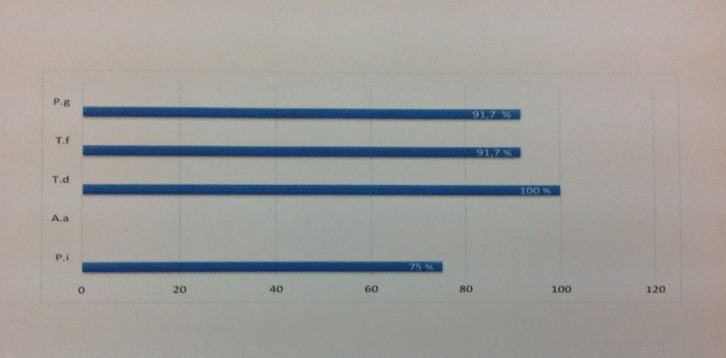
Frequency of detection of periodontopathogenic bacteria. *P. g: Porphyromonas gingivalis T. f: Tannerella forsythia T. d: Treponema denticola A. a: Aggregatibacter actinomycetemcomitans P. i: Prevotella intermedia*

All patients had at least one member of the red complex. *P. gingivalis* and T. forsythia were found in eleven of twelve patients whereas *T. denticola* was found in all of them. *P. intermedia* was found in proportion of 75%. But *A. actinomycetemcomitans* was no detected (Fig. 1). The association was strong between *P. gingivalis* and *T. forsythia* (*p*=0.001). *T. denticola* demonstrated a very strong association with *P. gingivalis*, *T. forsythia* and *P. intermedia* (*p*<0.001). *P. intermedia* was poorly correlated to *P. gingivalis* and *T. forsythia* (r=0.52). As *A. actinomycetemcomitans* was not detected in this pilot study, any association was not found ( Table 3).

**Table 3:**
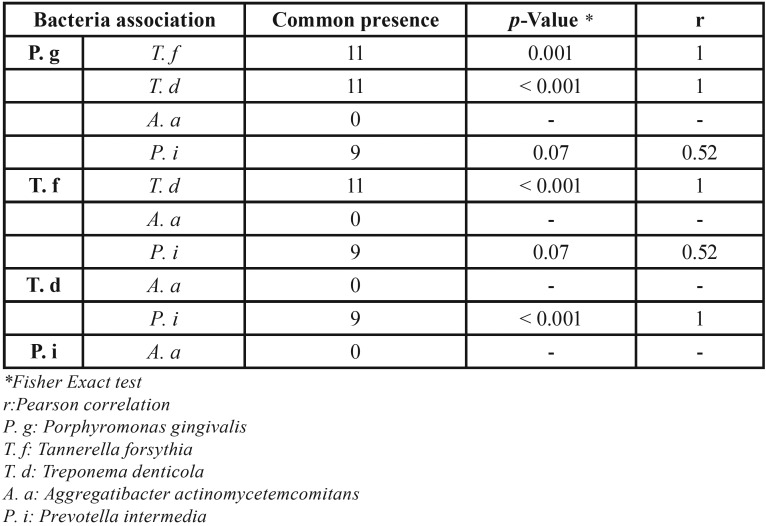
Association between different bacteria.

## Discussion

The objective of this pilot study was to assess the prevalence of five periopathogens in Congolese patients with periodontitis and to analyze the association among these five periopathogens. This study demonstrated a high prevalence of periopathogens, particularly the high prevalence of the red complex. This is the first study in DR Congo to screen for periopathogens in subgingival plaque of Congolese patients with periodontitis. According to the literature, the distribution and occurrence of periopathogenic bacteria vary depending on geographical locations, ethnic or race groups (9,15,16,17).

The results of this study demonstrated the high prevalence of *P. gingivalis*, *T. forsythia*, *T. denticola* and *P. intermedia*. This is in agreement with others studies that have found high prevalence of these bacteria in diseased sites (3,6,14). *A. actinomycetemcomitans* was not detected in this pilot study. It was reported that this bacterium was the key periodontal pathogen in aggressive periodontitis especially in localized aggressive periodontitis (9-11). Its presence in case of chronic periodontitis was also reported (18). In this study, the cases of chronic and aggressive periodontitis were included, but no stress was made at this point of view because of the small sample size. Others studies using culture and molecular techniques had detected this bacterium only at low prevalence (19,20).

*P. gingivalis* and *T. forsythia* were detected at high level of 92% and *T. denticola* in all patients. The presence of these three pathogens, belonging to the red complex described by Socransky *et al.* 1998, may be interpreted as a sign of disease progression. Indeed, a study by Byrne *et al.* has demonstrated that the levels of *P. gingivalis* and *T. denticola* could be used to predict disease progression (6). Patients included in this study had at least two teeth with PPD of at least 5mm and CAL of at least 4mm and the mean BoP was high. This high BoP may also be considered as a possible sign of the progression of disease (20).

Strong associations were found between three bacteria of the ‘”red complex”. Earlier studies have suggested the association between *P. gingivalis* (formerly Bacteroides gingivalis) and *T. forsythia* (formerly Bacteroides forsythus) (21), *P. gingivalis* and *T. denticola* (22-24). Describing the first relationship, Gmϋr *et al.* 1989 (21) concluded that this association was strong and *P. gingivalis* was not never found in the absence of *T. forsythia*. This is in agreement with the results of this study. In spite of the small size of this pilot study, it should be noticed that when *P. gingivalis* was absent, so was *T. forsythia*. The consensus report from the 1996 world workshop on clinical periodontology designated these two bacteria as etiological agents of chronic periodontitis (25). Their presence in subgingival plaque in increasing levels with increasing periodontal pockets led the participants to consider them to be the lead candidates in causing the progression of disease (25,6).

The relationship between *P. gingivalis* and *T. denticola* was stressed by other authors in animal models (26,27). In particular a study by Tan *et al.* suggested that these periopathogens grow synergistically by co-operating metabolically. Indeed, these authors showed, using continuous co-culture that *P. gingivalis* and *T. denticola* symbiotically co-exist and adapt each other by modulating gene expression, particularly the genes involved in metabolism and virulence (28).

The proteolytic enzymes capable of degrading host proteins and dysregulating the immune response are common to *P. gingivalis*, *T. forsythia* and *T. denticola* (6). These enzymes are thought to play an important role in the pathogenesis of periodontitis (29). Recently, *P. gingivalis* considered as a keystone pathogen of chronic periodontitis, was proposed to be detected by rapid chair-side test method (30). This method should be an adjunctive for the assessment of risk for chronic periodontitis and peri-implantitis.

The main shortcoming of this pilot study is the small size of the sample. Future studies involving large samples and comparing both microbial profiles of aggressive and chronic periodontitis and healthy controls in Congolese patients are needed. It would also be important to check for other periopathogenic taxa and communities using operational taxa units (OTUs).

## Conclusions

Within the limitations of this study, it provides for the first time data about periopathogens in the subgingival plaque of Congolese patients with periodontitis. The occurrence of the red complex is high and *A. actinomycetemcomitans* was not detected. *P. intermedia*, a member of the orange complex was frequently detected. Associations between *P. gingivalis*. and *T. forsythia*., *P. gingivalis* and *T. denticola* and *T. denticola* and *T. forsythia* were strong.
